# Characteristics of the Urinary Proteome in Women with Overactive Bladder Syndrome: A Case-Control Study

**DOI:** 10.3390/jcm10112446

**Published:** 2021-05-31

**Authors:** Marianne Koch, Pavel Lyatoshinsky, Goran Mitulovic, Barbara Bodner-Adler, Sören Lange, Engelbert Hanzal, Wolfgang Umek

**Affiliations:** 1 Department of Obstetrics and Gynecology, Medical University of Vienna, 1090 Vienna, Austria; barbara.bodner-adler@meduniwien.ac.at (B.B.-A.); soeren.lange@meduniwien.ac.at (S.L.); engelbert.hanzal@meduniwien.ac.at (E.H.); wolfgang.Umek@meduniwien.ac.at (W.U.); 2 Department of Urology, Cantonal Hospital St Gallen, 9007 St Gallen, Switzerland; p.liatochinski@gmail.com; 3 Clinical Institute of Laboratory Medicine and Proteomics Core Facility, Medical University of Vienna, 1090 Vienna, Austria; goran.mitulovic@meduniwien.ac.at; 4 Karl Landsteiner Society, Institute for Special Gynecology and Obstetrics, 3100 St. Pölten, Austria

**Keywords:** overactive bladder syndrome, mass spectrometry, proteomics, urine, pathophysiology, antimicrobial, apoptosis, cellular response to stress, protein pathway

## Abstract

Despite an estimated prevalence of 13% in women, the exact etiology of non-neurogenic overactive bladder syndrome is unclear. The aim of our study was to gain a better understanding of the pathophysiology of female overactive bladder syndrome by mapping the urinary proteomic profile. We collected urine samples of 20 patients with overactive bladder syndrome and of 20 controls. We used mass spectrometric analysis for label-free quantitation, Swissprot human database for data search, Scaffold for data allocation and the Reactome Knowledgebase for final pathway enrichment analysis. We identified 1897 proteins at a false discovery rate of 1% and significance level *p* < 0.001. Thirty-seven significant proteins of the case group and 53 of the control group met the criteria for further pathway analysis (*p* < 0.0003 and Log2 (fold change) >2). Significant proteins of the overactive bladder group were, according to the 25 most relevant pathways, mainly involved in cellular response to stress and apoptosis. In the control group, significant pathways mainly concerned immunological, microbial-protective processes and tissue- elasticity processes. These findings may suggest a loss of protective factors as well as increased cellular response to stress and apoptosis in overactive bladder syndrome.

## 1. Introduction

Non-neurogenic overactive bladder syndrome (OAB), defined as “urgency, with or without urge incontinence, usually with frequency and nocturia” is a very common disease in women [1]. Its estimated life-time prevalence is 13%, which increases up to 48% if associated with obesity. In women over 70 years of age, OAB reaches a peak-prevalence of 70% [2,3]. OAB causes a large socio-economic burden with high annual health care costs, but also a social impact on affected women including social isolation, loss of confidence and depression [4,5]. The exact causes and pathophysiologic mechanisms of OAB are still poorly understood, leaving us with less than optimal treatment options. Neuronal, myogenic and inflammatory processes have previously been discussed regarding their influence on OAB, with, however, limited results [6,7]. Inflammatory proteins, in particular neuronal growth factor, brain- derived neurotrophic factor, prostaglandins, cytokines, adipokines, monocyte chemoattractant protein-1 and C-reactive protein, have been described as significantly higher abundant in OAB patients compared to controls [8,9,10,11,12,13,14,15]. However, these inflammatory proteins are unspecific and elevated in other conditions as well, such as acute cystitis or urinary tract calculi. Moreover, the applied techniques for identification of these markers, such as enzyme-linked immunosorbent assay, require the use of specific and known antibodies for pre-specified proteins. Thus, only proteins already suspected to be involved in OAB will be detected, and the majority of proteins will not be investigated at all, although they might have a role in the development of OAB. In contrast, the proteomic technique employs chromatographic separation and mass spectrometry, and ultimately identifies and quantifies all proteins of a sample. This method provides a greater overview of the protein turnover in women with OAB in comparison to healthy controls. We have successfully used this approach previously, and were able to identify a urinary proteomic pattern in women with stress urinary incontinence [16,17]. Preliminary findings from a urinary proteomic study on OAB describe vascular cell adhesion protein (VCAM-1) in significantly different abundance in OAB patients compared to controls, but this study included only 16 samples [18]. Identification and comparison of the urinary proteome in patients with OAB should contribute to a more profound understanding of the etiology and could provide a basis for new treatment options. The objective of this study was therefore to identify a urinary proteomic pattern characteristic for OAB.

## 2. Materials and Methods

This prospective case-control study was conducted at the Department of Obstetrics and Gynecology in cooperation with the Core Facility Proteomics and the Clinical Department of Laboratory Medicine at the Medical University of Vienna between 2015 and 2020. The Institutional Review Board approval was obtained from the Ethics Committee of the Medical University of Vienna (no. 1376/2014). Written permission from all participants has been obtained. The inclusion criteria for the OAB group were: Females ≥18 years of age with symptoms of overactive bladder syndrome for ≥3 months (Zitat ICS). Exclusion criteria were: previous medical therapy for OAB within the previous 3 months; pregnancy, lactation, any neurologic conditions, pelvic organ prolapse (stage > 2); lower urinary tract surgery within the previous six months; history of interstitial cystitis or pain associated with OAB; recurrent urinary tract infections defined as >3/year; acute urinary tract infection; urinary retention (with or without required self-catheterization); stress urinary incontinence or mixed urinary incontinence; bladder cancer; renal failure and urinary tract stones. Participants in the control group had to be continent and free of any OAB symptoms, all other exclusion criteria applied as well. Cases were recruited at the urogynecology outpatient clinic at the Department of Obstetrics and Gynecology (Medical University of Vienna). Controls were recruited among outpatients and inpatients of the Department of Obstetrics and Gynecology. Controls were matched for age (+/− 5 years) and body mass index. The outcome of the study was the sequence coverage, abundance, and quantity of identified urinary proteins. Included participants were subjected to a detailed patient history, the ICIQ-OAB (International Consultation on Incontinence Questionnaire on OAB) questionnaire (OAB and control group) and a three- day bladder diary for documentation of fluid intake, micturition, frequency, incontinence episodes and nocturia (only OAB group). Clinical evaluation of participants with OAB symptoms included a standard gynecologic examination, cystometry and clinical stress test. We obtained one mid-stream, clean-catch urine sample from each participant at a single time point between 8 a.m. and 2 p.m., which was followed by immediate urine dip stick testing to exclude acute urinary tract infection. All urine samples were subsequently processed within 15 min after collection to avoid protein decomposition and autolysis. For protein precipitation, 2 mL urine of each sample were extracted and protein precipitation was conducted according to the internally modified Wessel-Flüge method [19] and all solvents were kept at −20 °C. All working steps were performed on ice and centrifugation in a cooled centrifuge at +4° Celsius. After careful removal of the resulting supernatant, the remaining protein pellet was dried in the air and later on dissolved in 200 μL of 50 mM triethylammonium bicarbonate at pH 8.5 (TEAB). Protein concentration within the solution was determined using the DeNovix nanodrop photometer. Subsequent protein digestion was performed according to Mitulovic et al. [20]. Upon tryptic digest, 30 μL of tryptic peptides were diluted with 20 μL of aqueous 0.1% TFA and 1 µg of digested protein was injected. Each sample was injected three times and peptides were separated using the nanoRSLC UltiMate 3000 HPLC system by ThermoFisher (Bremen, Germany) coupled to the Q Exactive Orbitrap mass spectrometer equipped with the nanospray electrospray source (Thermo Fisher Scientific, Germering, Germany). The separation was performed using mobile phases and the separation gradient as described in Koch et al. [16]. Every sample injection was followed by two blank runs with injections of TFE for removal of the possible sample remains in the injector or on the trap column, and prevention of carryover in the separation system. Mass spectrometry (MS) analysis was performed using the Q Exactive Plus mass spectrometer (Thermo Fisher Scientific) and the “top 20” method for MS/MS experiment; that is, the 20 most intensive ions from the MS scan were selected for tandem MS (MS/MS), single-charged ions were excluded from fragmentation, and detected ions were excluded for further fragmentation for 2 min after initial MS/MS fragmentation had been performed. The mass resolution of 70,000 was selected for MS at AGC set to 3E6, MS/MS resolution was set to 35,000 and AGC set to 1E5 scans. Fragmentation was performed using the HCD approach at a normalized collision energy of 30 eV. Additionally, before the MS, UV peptide detection at 214 nm was also performed, which also served as quality control for HPLC separation. Raw MS/MS files were analyzed using Proteome Discoverer 2.3 (ThermoFisher Scientific, Bremen, Germany) and searching the Swissprot human database using the following parameters: taxonomy: Homo sapiens; modifications: carbamidomethyl on C as fixed, carboxymethylation on M as a variable; peptide tolerance was set to 10 ppm and the MS/MS tolerance to 0.05 Da; trypsin was selected as the enzyme used and two missed cleavages were allowed; False discovery rate (FDR) was set to 1% and the decoy database search was used for estimating the FDR. Scaffold was used for data allocation and The Reactome Pathway Knowledgebase for final pathway enrichment analysis. The sample size calculation was based on the approach of Jung et al. [21] for two-sided paired t-tests with 20 individuals per group. With these sample numbers, an effect size of 0.9 standard deviations can be detected with 80% power when controlling the false discovery rate at 5%. For the sample size calculation, the proportion of differentially abundant proteins was assumed to be 5%. We applied independent t-test or chi-square test for demographic data analysis, considered a *p*-value of <0.001 as statistically significant and conducted all analyses using the SPSS Statistics software (IBM, Armonk, NY, USA, Version 25). For analysis of relative protein abundance and comparison between groups, the Fisher’s exact test with Benjamini-Hochberg multiple test correction at 1% false discovery rate and *p*-value < 0.001 was applied. This manuscript was structured according to the “Strengthening the Reporting of Observational Studies in Epidemiology (STROBE) guideline”.

## 3. Results

**Figure 1:**
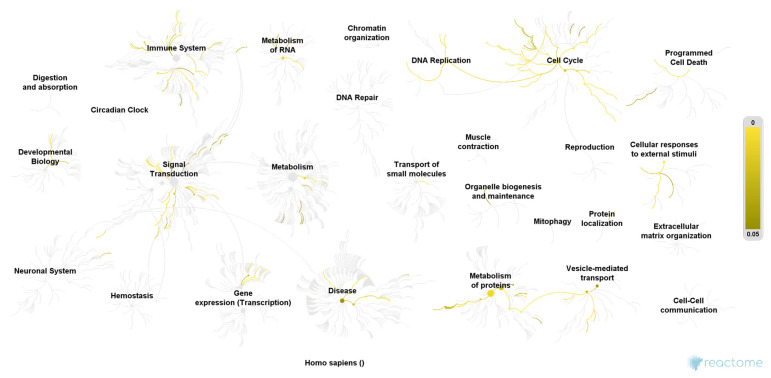
Pathway overview of the 27 most relevant proteins of the OAB group. Color codes indicate a hit in the respective pathway. Higher color intensity (yellow) means a higher significance of one or more proteins within this signal cascade (FDR). Reactome Pathway database, open access open source [22].

**Figure 2:**
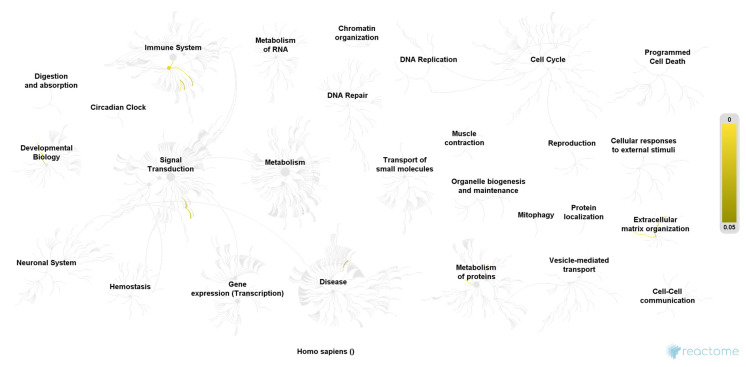
Pathway overview of the 35 most relevant proteins of the control group. Color codes (yellow) indicate a hit in the respective pathway. Higher color intensity (yellow) means a higher significance of one or more proteins within this signal cascade (FDR). Reactome Pathway database, open access open source [22].

We included 40 urine samples in the analysis (*n* = 20 cases and *n* = 20 controls). Demographic data were similar in both groups except for the ICIQ sum score (Table 1). We identified 1897 proteins in analyzed urine samples at a false discovery rate of 1% and the significance level of *p* < 0.001. Out of these, *297* proteins were found in significantly higher abundance in women with OAB as compared to controls (*p* < 0.003, Benjamini-Hochberg multiple test correction), of which 108 proteins were detected in the OAB group only. Furthermore, we found 269 proteins in significantly higher abundance in the control group (*p* < 0.0003, Benjamini-Hochberg multiple test correction), of which 111 proteins were exclusively detected among controls (Figure 1 and Figure 2). The remaining 1331 identified proteins were found in similar and non-significant abundance in both groups. Only proteins, which met the assigned criteria (significance level *p* < 0.0003 and Log2 (FC) > 2) were selected for pathway analysis, which resulted in 37 proteins of the OAB group (Table 2) and 53 proteins of the control group (Table 3). Pathway analysis was conducted using the Reactome Pathway Knowledgebase Enrichment Analysis (generated probability factor (*p*-value); Benjamini-Hochberg multiple test correction (FDR)). According to the resulting *p*-values, a ranking of the 25 most relevant signal cascades was generated for each group. Ten of the 37 most relevant proteins of the OAB group had to be excluded during pathway analysis as these could not be assigned to any pathway of the Reactome Pathway Knowledgebase. Similarly, 18 of the 53 most relevant proteins of the control group had to be excluded during pathway analysis. For the included 27 most relevant proteins of the OAB group, 386 different pathways were identified, each including a minimum of one identified protein. Similarly, for the included 35 most relevant proteins of the control group, 116 different pathways were identified, also each including a minimum of one identified protein. Table 4 and Table 5 show the 25 most relevant pathways of the OAB group and control group, respectively. A genome-wide overview of pathway analysis results is displayed in Figure 1 and Figure 2. Color codes indicate a hit in the respective pathway.

## 4. Discussion

To our knowledge, this is the largest study aiming to determine a urinary proteomic pattern in female overactive bladder syndrome for a better understanding of the diseases’ pathophysiology. We were able to identify significantly different protein pathways between women with OAB and healthy controls, suggesting a proteomic pattern characteristic for this disease. Several significant proteins of the OAB group could be assigned to the pathways “cellular responses to stress” and “regulation of apoptosis”, which may suggest a disruption of normal homeostasis by external stimuli. The ability of cells to respond to external influences, and to sustain a fine balance between cell survival and apoptosis, is crucial for normal development and homeostasis [24]. Disruption of this balance can contribute to the development of autoimmune diseases, neurodegeneration, and cancer [25]. In contrast, the most relevant pathways of the control group belong to the categories “extracellular matrix organization” and “immune system”, which suggests the presence of a more protective immune system and a generally healthier tissue turnover of the urinary bladder. Our findings may indicate a disruption in the capability of the urinary bladder tissue to adequately respond to external stimuli in OAB patients—possibly due to an altered immune response. One smaller proteomic study on OAB identified the protein VCAM-1 as only significantly different protein when compared to controls [18]. VCAM-1 protein plays a role in immune response and leukocyte emigration and can be located in the pathway categories “extracellular matrix organization” and “immune system”. Even though VCAM-1 was not identified as significant protein in our analysis, several significant protein pathways of our analysis could be assigned to the same categories. In congruence with results from this prior study, we also suggest alterations in the immune system- among others- as relevant in the pathophysiology of OAB. Surprisingly, further significant proteins of the OAB group are involved in neuronal processes (Spectrin beta chain, non-erythrocytic 2; 3-hydroxyacyl-CoA Dehydrogenase type 2; Cytoplasmic dynein 1 heavy chain 1; Isoform 2 of glutamine--tRNA ligase; Isoform 2 of Nck-associated Protein 1). Subsequent pathway analysis substantiated this finding where the Asparagine N-linked glycosylation signal cascade was identified as one of the 25 most relevant pathways in the OAB group. N-linked glycosylation is a form of post-translational modification and most important for proteins synthesized and folded in the endoplasmic reticulum [26]. Mutations in the relevant genes may trigger severe developmental problems which are frequently involving the central nervous system [27]. Our study population was carefully selected to guarantee only non-neurogenic OAB cases. Thus, the detection of significant proteins linked to neurological processes in our OAB group came somewhat unexpected. If confirmed, this could indicate a neurogenic component, even in the pathophysiology of non-neurogenic OAB. Hypersensitivity of the urinary bladder, which clinically shows as frequency and urgency, may be explained by such alterations in neurological processes. Overall, we were able to observe a larger diversity of pathways and their categories in women with OAB compared to controls, which is displayed in Figure 1 and Figure 2. Whereas significant protein pathways of both case and control groups appear in the same category (e.g., the category “immune system”), it seems interesting that the respective pathways do not overlap between the groups. This underlines the uniqueness of the urinary proteome of women with OAB. Strengths of this study are the very strict inclusion criteria of the study population. We included women with pure OAB symptoms only and no other form of incontinence or lower urinary tract symptoms. The observed differences in the urinary proteome can, therefore, be fully attributed to the presence or absence of OAB symptoms. In contrast to previous studies, we were able to identify and compare the complete proteome of urine samples, which allows a more holistic view of the pathophysiology of OAB. While other studies focused on proteins, already imagined of with a role in the development of OAB (mainly inflammatory proteins), we took a step back and looked at the complete proteome, being able to identify previously unsuspected compounds. Using this approach, previously neglected but important molecular processes characteristic for OAB, can be drawn to attention. We also have to acknowledge certain limitations of this study. It can be assumed that the urinary proteome follows a circadian rhythm as well as changes according to food and drink intake [28]. As we only took one urinary sample per participant at one time point, we are unable to account for this intra-individual proteome turnover. However, as cases and controls equally underlie this circadian proteome rhythm, we assume that our observed differences should nevertheless be valid. Another limitation is the yet incomplete human proteome mapping, meaning that global research has not yet been able to identify and describe all proteins of the human body. Results from our study can therefore only reflect the status quo of proteome knowledge. 

## 5. Conclusions

We found a more diverse protein turnover in urine samples of the OAB group compared to controls. Significant pathways describe protein interactions at different time points of the cell cycle and processes of phosphorylation and degradation, as well as regulation of cellular response to stress. Unexpectedly, we found several significant proteins and pathways involved in neurological processes, which may indicate a neurogenic component even in non-neurogenic OAB. Significant protein pathways of the control group mainly concerned immunological, microbial-protective processes, and tissue-elasticity processes. We suggest a combination of increased cellular response to stress and apoptosis, as well as a loss of protective factors (such as antimicrobial and tissue-elasticity) as characteristic for OAB.

## Figures and Tables

**Table 1:** Demographic data.

	OAB (*N* = 20)	Control (*N* = 20)	*p*-Value
(Mean ± SD)			
Age (years)	57.5 (±16)	55.3 (±17)	0.904
BMI (kg/m^2^)	27.7 (±5)	25.5 (±5)	0.740
Vaginal deliveries (*n*)	1.15	0.99	0.484
Chronic diseases * (*n*)	12/20 (60%)	8/20 (40%)	0.206
Menopausal status (*n*)			0.744
premenopausal	7/20 (35%)	8/20 (40%)	
postmenopausal	13/20 (65%)	12/20 (60%)	
Smoking (yes)	2/20 (10%)	3/20 (15%)	0.633
Caffeine consumption	17/20 (85%)	16/20 (80%)	0.677
ICIQ sum score	31	0	-

* at least one of the following: hypertension, coronary heart disease, bronchitis, glaucoma, depression, irritable bowel syndrome, COPD, DM type 2, adipositas, hyperlipidemia, hypercholesterolemia.

**Table 2:** Significant proteins detected in the overactive bladder syndrome group.

Protein	Accession	Gene Symbol
26S proteasome non-ATPase regulatory subunit 11	O00231	PSD11
26S proteasome regulatory subunit 10B	A0A087 × 2I1	A0A087 × 2I1
3-hydroxyacyl-CoA dehydrogenase type-2	Q99714	HCD2
60S ribosomal protein L15	P61313	RL15
Alpha-centractin	R4GMT0	R4GMT0
Alpha-mannosidase 2	Q16706	MA2A1
Asparagine--tRNA ligase, cytoplasmic	O43776	SYNC
Beta/gamma crystallin domain-containing protein 1	A0A0J9YWL0	A0A0J9YWL0
Bleomycin hydrolase	Q13867	BLMH
Coatomer subunit beta	P53618	COPB
Cytoplasmic dynein 1 heavy chain 1	Q14204	DYHC1
Cytosolic phospholipase A2 delta	Q86XP0	PA24D
Electron transfer flavoprotein subunit alpha, mitochondrial	P13804	ETFA
Eukaryotic translation initiation factor 2 subunit 1	P05198	IF2A
Eukaryotic translation initiation factor 3 subunit A	Q14152	EIF3A
Heat shock 70 kDa protein 4L	O95757	HS74L
Isoform 2 of 26S proteasome regulatory subunit 6B	P43686-2	PRS6B
Isoform 2 of Alpha-S1-casein	P47710-2	CASA1
Isoform 2 of Glutamine--tRNA ligase	P47897-2	SYQ
Isoform 2 of Nck-associated protein 1	Q9Y2A7-2	NCKP1
Isoform 2 of Serine/threonine-protein phosphatase 2A 55 kDa regulatory subunit B alpha isoform	P63151-2	2ABA
Isoform 2 of Threonine--tRNA ligase, cytoplasmic	P26639-2	SYTC
Kappa-casein	P07498	CASK
Mitogen-activated protein kinase 1	P28482	MK01
Mucin-4	Q99102	MUC4
Peroxisomal acyl-coenzyme A oxidase 1	Q15067	ACOX1
Polypyrimidine tract-binding protein 1	A0A0U1RRM4	A0A0U1RRM4
Prenylcysteine oxidase 1	Q9UHG3	PCYOX
Proteasome subunit alpha type-2	P25787	PSA2
Protein flightless-1 homolog	Q13045	FLII
Spectrin beta chain, non-erythrocytic 2	O15020	SPTN2
Spliceosome RNA helicase DDX39B	Q13838	DX39B
Tripartite motif-containing protein 16	O95361	TRI16
Tropomodulin-3	Q9NYL9	TMOD3
UDP-glucose 4-epimerase	Q14376	GALE
Vesicle-fusing ATPase	P46459	NSF
X-ray repair cross-complementing protein 5	P13010	XRCC5

**Table 3:** Significant proteins detected in the control group.

Protein	Accession	Gene Symbol
Trefoil factor 3	X6R3S7	X6R3S7
Latent-transforming growth factor beta-binding protein 2	G3V3 × 5	G3V3 × 5
Meprin A subunit	B7ZL91	B7ZL91
Testican-1	A0A0A0MQX7	A0A0A0MQX7
IgGFc-binding protein	Q9Y6R7	FCGBP
Frizzled-4	Q9ULV1	FZD4
Mucin-5B	Q9HC84	MUC5B
Growth/differentiation factor 15	Q99988	GDF15
Isoform 2 of Phosphoinositide-3-kinase-interacting protein 1	Q96FE7-2	P3IP1
BPI fold-containing family B member 1	Q8TDL5	BPIB1
CD99 antigen-like protein 2	Q8TCZ2	C99L2
Adhesion G protein-coupled receptor F5	Q8IZF2	AGRF5
Protein S100-A7A	Q86SG5	S1A7A
Elongation factor Tu	Q83ES6	EFTU
Protocadherin Fat 4	Q6V0I7	FAT4
Na(+)/H(+) exchange regulatory cofactor NHE-RF3	Q5T2W1	NHRF3
FRAS1-related extracellular matrix protein 2	Q5SZK8	FREM2
Trefoil factor 2	Q03403	TFF2
Fibulin-2	P98095	FBLN2
Hepcidin	P81172	HEPC
Brain acid soluble protein 1	P80723	BASP1
Hemoglobin subunit gamma-2	P69892	HBG2
NPC intracellular cholesterol transporter 2	P61916	NPC2
Beta-defensin 1	P60022	DEFB1
Secreted Ly-6/uPAR-related protein 1	P55000	SLUR1
Isoform 2 of Cysteine-rich secretory protein 3	P54108-2	CRIS3
Afamin	P43652	AFAM
Fibrillin-1	P35555	FBN1
Isoform 2 of Syndecan-4	P31431-2	SDC4
Granulins	P28799	GRN
Serum paraoxonase/arylesterase 1	P27169	PON1
CD27 antigen	P26842	CD27
Fibulin-1	P23142	FBLN1
Small proline-rich protein 2D	P22532	SPR2D
Cornifin-B	P22528	SPR1B
Azurocidin	P20160	CAP7
Elafin	P19957	ELAF
Inter-alpha-trypsin inhibitor heavy chain H2	P19823	ITIH2
Complement component C7	P10643	CO7
Matrilysin	P09237	MMP7
Tumor necrosis factor receptor superfamily member 16	P08138	TNR16
Calbindin	P05937	CALB1
Heparin cofactor 2	P05546	HEP2
Major prion protein	P04156	PRIO
Trefoil factor 1	P04155	TFF1
Antileukoproteinase	P03973	SLPI
Cystatin-A	P01040	CYTA
Complement C5	P01031	CO5
Plasminogen	P00747	PLMN
Superoxide dismutase [Cu-Zn]	P00441	SODC
CD5 antigen-like	O43866	CD5L
Ribonuclease T2	O00584	RNT2
Isoform 2 of Deoxyribonuclease-2-alpha	O00115-2	DNS2A

**Table 4:** Pathway analysis of most relevant proteins of the OAB group (ranked according to the *p*-value of the enrichment analysis of the Reactome Pathway Analysis of the OAB group; modified after Fabregat et al. [23]).

Pathway	Ratio of Identified Proteins	*p*-Value	False Discovery Rate
COPI-mediated anterograde transport	5/102	0.00003	0.006
Transport to the Golgi and subsequent modification	6/186	0.00004	0.007
ER to Golgi Anterograde Transport	5/155	0.0002	0.012
ABC-family proteins mediated transport	4/103	0.0005	0.012
Cellular responses to stress	7/408	0.0005	0.012
G2/M Transition	5/198	0.0006	0.012
Asparagine N-linked glycosylation	6/305	0.0006	0.012
Mitotic G2-G2/M phases	5/200	0.0007	0.012
Regulation of activated PAK-2p34 by proteasome mediated degradation	3/50	0.0007	0.012
Cross-presentation of soluble exogenous antigens (endosomes)	3/50	0.0007	0.012
Intra-Golgi and retrograde Golgi-to-ER traffic	5/206	0.0007	0.012
Regulation of ornithine decarboxylase (ODC)	3/51	0.0008	0.012
Autodegradation of the E3 ubiquitin ligase COP1	3/52	0.0008	0.012
Ubiquitin-dependent degradation of Cyclin D1	3/52	0.0008	0.012
Ubiquitin-dependent degradation of Cyclin D	3/52	0.0008	0.012
Transcriptional regulation by RUNX2	4/121	0.0009	0.012
Vpu mediated degradation of CD4	3/53	0.0009	0.012
Regulation of Apoptosis	3/53	0.0009	0.012
p53-Independent DNA Damage Response	3/53	0.0009	0.012
p53-Independent G1/S DNA damage checkpoint	3/53	0.0009	0.012
Ubiquitin Mediated Degradation of Phosphorylated Cdc25A	3/53	0.0009	0.012
FBXL7 down-regulates AURKA during mitotic entry and in early mitosis	3/55	0.0010	0.012
SCF-beta-TrCP mediated degradation of Emi1	3/55	0.0010	0.012
Degradation of AXIN	3/55	0.0010	0.012
Negative regulation of NOTCH4 signaling	3/55	0.0010	0.012

**Table 5:** Pathway analysis of most relevant proteins of the control group (ranked according to the *p*-value of the enrichment analysis of the Reactome Pathway Analysis of the OAB group; modified after Fabregat et al. [23]).

Pathway	Ratio of Identified Proteins	*p*-Value	False Discovery Rate
Molecules associated with elastic fibers	4/38	0.00004	0.004
Elastic fiber formation	4/45	0.00007	0.004
Terminal pathway of complement	2/8	0.0007	0.026
Antimicrobial peptides	4/95	0.001	0.033
Regulation of Insulin-like Growth Factor (IGF) transport and uptake by Insulin-like Growth Factor Binding Proteins (IGFBPs)	4/124	0.003	0.054
Extracellular matrix organization	6/301	0.003	0.054
Formation of the cornified envelope	4/129	0.003	0.054
Innate Immune System	12/1183	0.009	0.122
Activation of Matrix Metalloproteinases	2/33	0.011	0.132
NFG and proNGF binds to p75NTR	1/3	0.014	0.132
Ceramide signaling	1/3	0.014	0.132
Post-translational protein phosphorylation	3/107	0.015	0.132
Axonal growth stimulation	1/4	0.019	0.151
Keratinization	4/217	0.020	0.160
NADE modulates death signaling	1/6	0.028	0.169
p75NTR negatively regulates cell cycle via SC1	1/6	0.028	0.169
Metal sequestration by antimicrobial proteins	1/6	0.028	0.169
Degradation of the extracellular matrix	3/140	0.029	0.176
Activation of C3 and C5	1/7	0.033	0.187
RNF mutants show enhanced WNT signaling and proliferation	1/8	0.037	0.187
Axonal growth inhibition (RHOA activation)	1/9	0.042	0.205
Synthesis of 5-eicosatetraenoic acids	1/9	0.042	0.205
p75NTR regulates axonogenesis	1/10	0.047	0.205
Regulated proteolysis of p75NTR	1/11	0.051	0.205
Dissolution of Fibrin Clot	1/13	0.06	0.224

## Data Availability

The data presented in this study are available on request from the corresponding author.
